# Evaluating the influence of various friction stir processing strategies on surface integrity of hybrid nanocomposite Al6061

**DOI:** 10.1038/s41598-024-58714-3

**Published:** 2024-04-05

**Authors:** Navid Molla Ramezani, Behnam Davoodi

**Affiliations:** School of Mechanical Engineering, Iran University of Science and Technology, Tehran, Iran

**Keywords:** Friction stir processing (FSP), Surface integrity, Hybrid composite, FSP strategies, Metal matrix composite, Mechanical engineering, Nanoparticles

## Abstract

To fundamentally investigate the influence of different friction stir processing (FSP) strategies, namely raster, spiral, and parallel in various passes on the surface integrity of hybrid aluminum nanocomposites reinforced by titanium oxide (TiO_2_), silicon carbide (SiC), and zirconium oxide (ZrO_2_) nanoparticles, various examinations were conducted. The surface integrity, comprising microstructural characterization, elemental composition, surface topography, roughness, waviness, and microhardness was studied by different analyses, including scanning electron microscopy (SEM), energy-dispersive X-ray spectroscopy (EDS), optical microscopy (OM), atomic force microscopy (AFM), and Vickers microhardness machine in different zones. Results demonstrated that surface integrity and quality are dependent on the type of FSP strategy. SEM images revealed that a homogeneous distribution of the nanoparticles in the matrix is obtainable by the parallel and raster FSP strategies. Roughness and waviness measurements illustrated that the surface topography of the hybrid nanocomposite was symmetrical and improved by raster strategy and TiO_2_ + ZrO_2_ nanoparticle reinforcement. Furthermore, the two-pass FSP improved the arithmetic average surface value (R_a_) such that the R_a_ of two passes decreased by 32.5% compared to a single one. The mean microhardness in the spiral, raster, and parallel pass strategies increased by ~ 45%, 37%, and 31%, respectively.

## Introduction

Recently, researchers developed hybrid nanocomposites and composites to acquire the unique advantages of nanoparticles and reinforcements simultaneously and overcome the limitations of mono-composites^1,2^. Focusing on one or at most two intended properties could be considered as the main drawback of mono-composites and mono-nanocomposites^3,4^; therefore, hybrid metal matrix composites (HMMC) and hybrid metal matrix nanocomposites (HMMNC) are developed with different reinforcements and particles to improve their mechanical properties and surface integrity, as well as enhancing any property of each composite, depending on the added particle type^5^.

Surface integrity is a complete definition of the HMMC and HMMNC quality, including surface topography, metallurgical surface, and mechanical properties. Thus, a thorough comprehension of the effect of friction stir processing (FSP) on nanocomposites is essential for many industrial applications^6^. Surface topography and morphology include waviness and surface roughness. On the one hand, hybrid nanocomposites with grate surface morphology permit additional industrial applications for pieces undergoing high force, temperature, cycle loading, and friction conditions. On the other hand, due to the potential reinforcement matrix phase pullout, surface nanocomposite fabricated by FSP could lead to unfavorable waviness and roughness^7^. Surface metallurgy includes physical, chemical, metallurgical, recrystallization, and phase transformation on the friction stir processed surface and subsurface. Finally, the mechanical characteristics of hybrid nanocomposites after processing included variations of micro-hardness and residual stresses (generally quantified a profile in composite depth by micro-indentation and XRD)^8^.

**Figure 1:**
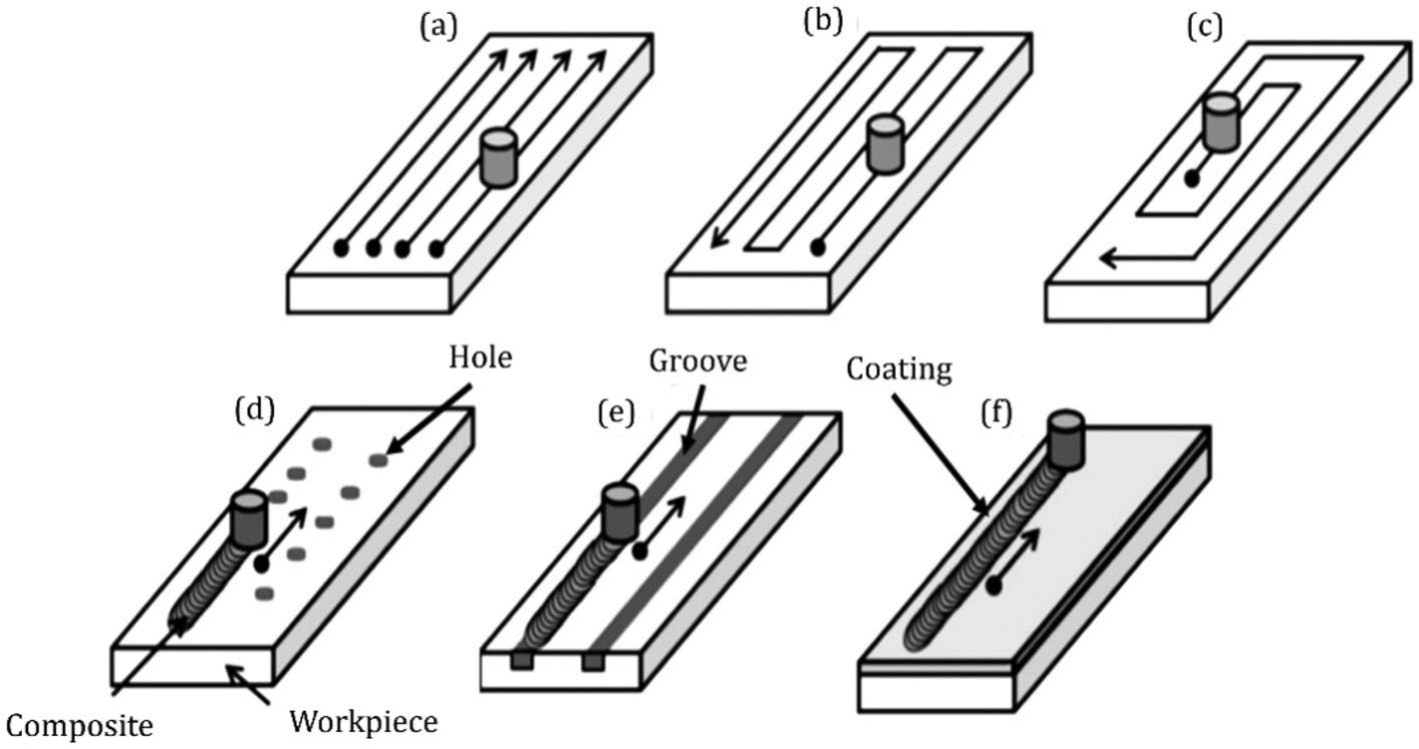
Schematic of friction stir processing tool path and reinforcement strategies^17^: (**a**) parallel tool path (**b**) raster tool path (**c**) spiral tool path (**d**) hole reinforcement strategy (**e**) groove reinforcement strategy, and (**f**) coating reinforcement strategy.

FSP is one of the novel and successful solid-state techniques for creating and improving the surface integrity of hybrid nanocomposites^9^. Hybrid nanocomposite fabricated by FSP is the preferred procedure for producing nanocomposite because it is an efficient and safe choice for reaching homogenized nanocomposites^10,11^. The most challenging section of this process is selecting the suitable parameters^12^. FSP parameters are mainly dependent on many variables, such as machine parameters, tool parameters, and material properties^13,14^. The tool path strategy of the friction stir processing is one of the principal machine parameters. The FSP strategy remarkably influences the surface integrity and mechanical properties^15^. Producing defect-free and uniform nanoparticles is challenging due to asymmetrical material flow during the thermochemical process that could be decreased by changing the tool path. Two strategies to expand the width of the surface composite by FSP are available^16^. The first is to use a tool with a considerable diameter, and the second is to process using multi-pass FSP. The large FSP tool dimension causes an increase in the required force and power consumption of the FSP machine. Multi-pass FSP with a proper overlap can ensure the demanding surface quality of the metal matrix composite^17^. FSP strategies, such as raster, spiral, and parallel passes are procedures for generating paths that optimize FSP efficiency and guarantee the surface quality of composites (Fig. 1). FSP strategy resolution is nearly related to surface integrity, whereas tool path orientation specifically affects the processing time. The theoretical FSP time for wide surface composite is obtained by totaling each path length feature by its traverse speed. It should be noted that this time underestimates the real-time because the computation neglects the deceleration and acceleration of the machine's effects^18^. The strategy generally used in FSP is the parallel path strategy. The parallel path strategy is simple to generate, but this path is discontinuous. Furthermore, there is adequate time for the cooling in the revolution period, hence decreasing the tool wear^19^. On the other hand, with the raster and spiral strategy, the tool path is continuous and appropriate for high-speed manufacturing^20^.

Reinforcement nanoparticles are inserted into the metal matrix in various techniques during FSP^10^. Many papers and research have attempted to introduce nanoparticles of reinforcement with different strategies. Most of the researchers studied FSP straight pass. Besides, they selected the direction of the groove perpendicular to the traverse speed and confirmed that the particles could better blend with the metal matrix^21–27^. In addition, the microstructure of mono-composites compared with hybrid-composites is more complicated. Uniformly distributed reinforcement in the matrix straight affects the grain refinement. Also, agglomeration growth due to the diverse nanoparticle properties is critical to preparing uniform reinforcement in the stir zone^28–31^.

Kumar et al.^32^ investigated the effect of tool overlap and tool direction on the microstructure and defects of the FSP circular sample. The results indicated that the direction of tool rotation has a notable effect on defect formation and retention upon FSP of magnesium alloy with spiral FSP strategy. There is a direct relation between tool shoulder overlap, plastic deformation, and temperature. Besides, a higher tool improves second-phase dissolution and grain refinement while improving the hardness. Samanta et al.^33^ evaluated the effect of tool design on tensile properties and FSP of high-pressure die-cast heterogeneous microstructure of the A380 alloy process zone. In addition, FSP single pass and multi-pass with different orientations to create a defect-free zone were investigated. They found that tool design and a specific pass orientation could produce a uniform microstructure and improve tensile properties. Ramezani et al.^6,34^ investigated the surface integrity and mechanical properties of the Aluminum A7075 matrix mono nanocomposite. They studied the effects of machine parameters on surface roughness, topography, and microhardness by response surface methodology. Results showed pass number and traversing speed are the most significant parameters on surface quality and microhardness. Rathee et al.^35^ reviewed the effects of various tool offsets and reinforcement particle strategies on single-pass FSP of the AA6063 for the determined parameters. They concluded that the most significant parameters on homogeneity distribution were related to the position of the tool offset. The best result for powder distribution and considerable stir zone was with a 1.5 mm tool offset. Additionally, they found the groove method with tool offset showed better homogeneity in different patterns. Sharma et al.^36^ studied the aluminum alloy composites with silicon carbide particles fabricated by different processes, including tool offset overlap and dual-tool. Processed material flow indicated that the distribution of particles changed by the probe stirring action due to the movement of the tool. Moreover, results exhibited that the surface metal matrix composite by the larger diameter of the FSP tool creates a particle heterogeneous distribution and defects. Sharma et al.^37^ investigated various particle strategies of hybrid AA6061 composite by hole and groove procedure. They reported that friction stir processing of aluminum composites is influential due to noble tribology aspects. The hard ceramics lubricant reinforcement improves the hybrid composites' tribological parameters. Also, the hole procedure revealed better wear resistance due to an improved homogeneity of nanoparticles distribution. Moreover, FSP without nanoparticles showed weak wear resistance and hardness due to the strengthening precipitate loss. In the other study, Sunil^38^ reported that the secondary phase amount and the level of dispersion during FSP depend on tool design. Holes filling, groove filling, sandwiching, direct FSP tool method, and surface coating by FSP were employed to combine the secondary phase into the surfaces. In addition, hole or groove filling and then closing groove or holes with a pin-less tool before the process was presented as the optimum method to disperse more secondary phases.

As mentioned, surface composites and nanocomposites in different dimensions for various applications are increasingly being developed and expanded. Nevertheless, composites production with different materials and scales by friction stir processing, usually have been studied and researched on a limited level and dimensions (limited to tool diameter). Therefore, in this research, using different paths and different strategies, surface nanocomposites with high surface integrity and quality were studied and investigated to be used in high-performance applications. In such a way that, so far, no research has studied the strategy and tool paths for the production and development of nanocomposite by FSP. Furthermore, the distribution and homogeneity of nanoparticles in the base material depend on different and extensive parameters, and one of the most vital parameters of the process is the strategy and tool motion path, which has not been investigated in any research.

To the best knowledge of the authors, no experimental research has been reported on FSP strategies and tool path effect on surface integrity of cold rolled Al6065 hybrid nanocomposite fabricated by FSP. In the current study, different novel FSP strategies and various tool path profiles, including raster, spiral, and parallel passes were investigated. For a detailed study, all parameters and process variables are constant during experiments. Hybrid aluminum matrix nanocomposites consisting of a mixture of two and three various nanoparticles were processed. Finally, the surface integrity of processed samples, including surface topography, metallurgical surface, and mechanical properties was investigated and compared. For this purpose, microhardness, microstructure, energy-dispersive X-ray spectroscopy (EDS), surface roughness and waviness, and surface topography were investigated.

## Materials and methods

### Materials and procedure of friction stir processing

A sheet of aluminum A6061 alloy with a length of 500 mm, width of 150 mm, and thickness of 10 mm was used as the matrix for FSP. The nominal range of element composition of the matrix in weight percentage is presented in Table 1 (according to ASTM E406-81). A cylindrical FSP tool of tungsten carbide was chosen. The tool geometry was a shoulder with a diameter of 18 mm and a taper pin root with a diameter of 6 mm.

**Table 1:** Chemical composition of the metal matrix (wt%).

Elements	Al	Mg	Si	Fe	Cu	Mn	Cr	Zn
wt%	Base	0.8	0.5	0.5	0.15	0.1	0.05	0.03

**Figure 2:**
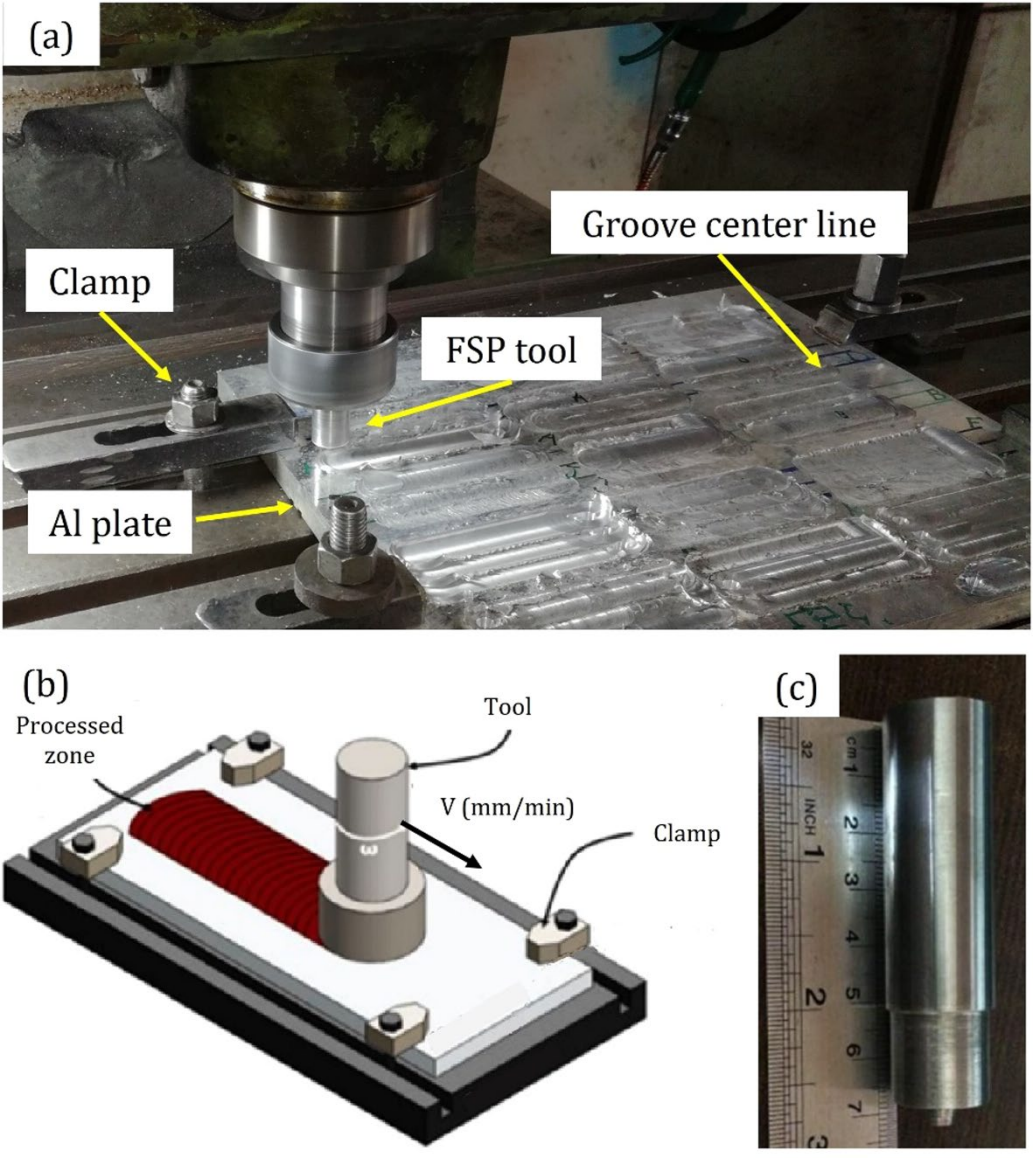
The friction stir processing: (**a**) experimental setup, (**b**) schematic of the process, adapted under license from ^3^ and (**c**) FSP tool.

The tool rotated clockwise (CW) during different FSP strategies with complete overlap. In addition, according to the literature review and experimental research, machine parameters (rotational speed of 1500 rpm and traverse speed of 40 mm/min) were constant for all experiments^6,35^. The experiments were repeated in two FSP passes to evaluate the effect of the pass number. The processing parameters and FSP setup with its schematic representation are presented in Table 2 and Fig. 2. Besides, nano titanium oxide (TiO_2_) particles, nano zirconium oxide (ZrO_2_) particles, and nano silicon carbide (SiC) particles (from the US Nano) with the size of 45–65 nm and purity of 99% used as reinforcement.

**Table 2:** The processing parameters in terms of strategy and nanoparticles during various FSP conditions with different passes.

Single FSP pass			Two FSP passes		
Sample No	Strategy	Nanoparticles	Sample no	Strategy	Nanoparticles
1	Parallel	TiO_2_ + ZrO_2_	13	Parallel	TiO_2_ + ZrO_2_
2	Parallel	TiO_2_ + SiC	14	Parallel	TiO_2_ + SiC
3	Parallel	ZrO_2_ + SiC	15	Parallel	ZrO_2_ + SiC
4	Parallel	Triple	16	Parallel	Triple
5	Raster	TiO_2_ + ZrO_2_	17	Raster	TiO_2_ + ZrO_2_
6	Raster	TiO_2_ + SiC	18	Raster	TiO_2_ + SiC
7	Raster	ZrO_2_ + SiC	19	Raster	ZrO_2_ + SiC
8	Raster	Triple	20	Raster	Triple
9	Spiral	TiO_2_ + ZrO_2_	21	Spiral	TiO_2_ + ZrO_2_
10	Spiral	TiO_2_ + SiC	22	Spiral	TiO_2_ + SiC
11	Spiral	ZrO_2_ + SiC	23	Spiral	ZrO_2_ + SiC
12	Spiral	Triple	24	Spiral	Triple

**Figure 3:**
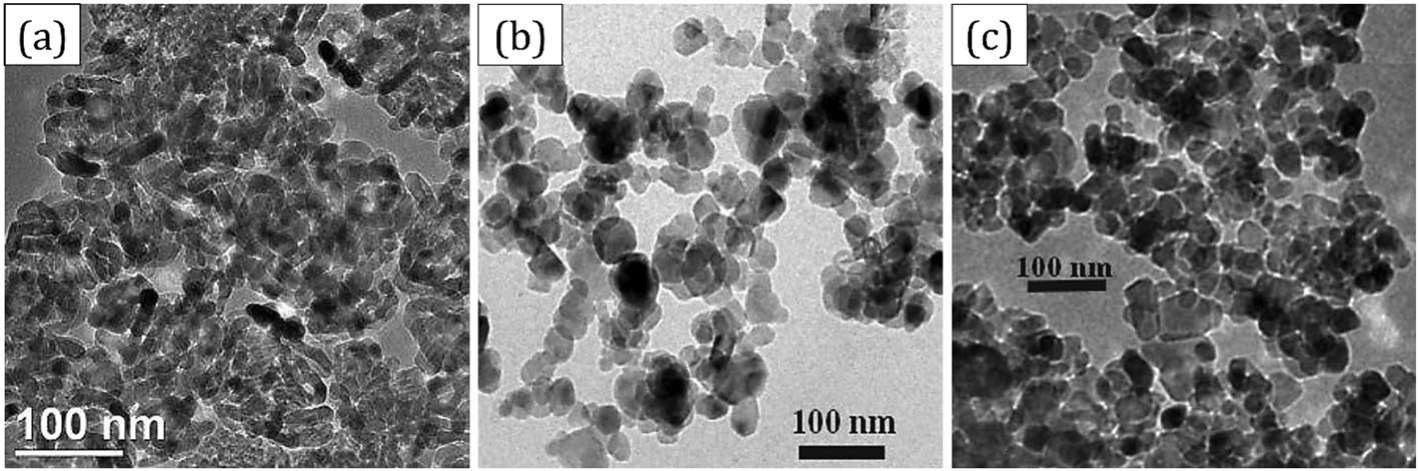
TEM images of nanoparticles: (**a**) titanium oxide (TiO_2_), (**b**) silicon carbide (SiC), and (**c**) zirconium oxide (ZrO_2_).

Figure 3 shows transmission electron microscopy (TEM) images of nanoparticles. The groove method was selected to replace the nanopowders. Grooves were machined with a depth of 3 mm and a width of 2 mm by an endmill tool. After the complete cleaning of grooves with acetone liquid and burr removal, they were filled with nanopowders and compressed. Before the FSP, the capping processes without the pin FSP tool were done. Then, the FSP processes were conducted by different tool paths to distribute reinforcement in the matrix. The agglomerated nanoparticles were dispersed by ultrasonic vibration for 20 min. Hybrid nanopowder and triple nanopowder were mixed in a “Y-cone powder mixer” for 4 h. The combination fraction of hybrid nanoparticles was placed (TiO_2_ + ZrO_2_, TiO_2_ + SiC, and ZrO_2_ + SiC) in the range of 50 wt%. Also, the combination fraction of triple nanoparticles was selected (Tio_2_ + ZrO_2_ + SiC) nanoparticles in 33.33 wt%.

### Surface topography

In this paper, two measurement techniques were employed to investigate surface topography: electronic measurement and scanning probe method. The displacement or profiling type of electronic measurement was used to measure the surface texture. The vertical movement is amplified electrically on a recorder surface profile provided as an average to give a number describing the surface roughness value. The sample's surface roughness with the R_a_ mechanism was measured by the Mahr-Perthometer M2 surface roughness meter. The roughness value was calculated in three paths with five-millimeter lengths, and then the average value was reported. Furthermore, in this procedure, the tip of the diamond stylus traveled across the sample surface at a continuous and constant speed. Moreover, the surface topography of nanocomposites was investigated by atomic force microscopy (AFM). The surface topography was measured to a fine scale, until the molecular grade. Also, the texture of the surface was obtained by monitoring the probe movement over the scanned surface.

### Microstructural examinations

**Figure 4:**
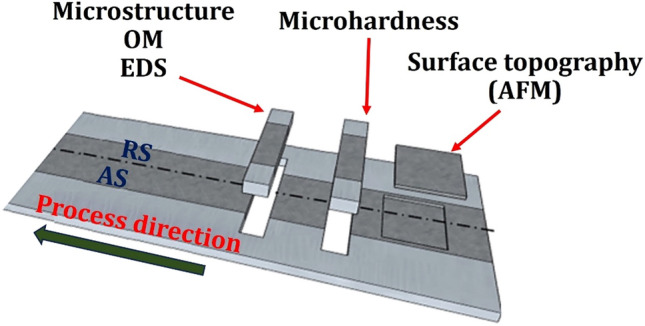
Schematics illustration of sample locations for determining surface roughness, surface topography, microstructure, and microhardness. Adapted under license from^39^.

The microstructure of HMMNCs was investigated by different analyses including scanning electron microscopy (SEM), energy-dispersive X-ray spectroscopy (EDS), and optical microscopy (OM). All samples and specimens were cross-sectioned perpendicular to the process direction using nontraditional spark machining (Fig. 4). The electro-etching was conducted to demonstrate the structure after grinding and polishing the specimens.

### Mechanical properties

The microhardness of HMMNCs was measured on a cross-section along the line 2 mm below the specimen surface by the Vickers microhardness in different microstructure zones. The microhardness measurement was done by applying a delay state of 15 s and a load of 50 gr (according to the ASTM E384). In addition, the surface microhardness of different strategies and reinforcements was investigated by the Vickers machine. Also, the profile of microhardness and the mean indentation microhardness for each sample zone were reported.

## Results and discussion

### Surface topography

The surface integrity of surface composites and nanocomposites fabricated by friction stir processing is a key parameter. Surface roughness and waviness are the main parameters that improve the surface integrity of the workpieces during different manufacturing processes^40,41^. The products with high surface quality have proper performance, lifespan, and mechanical properties such as fatigue life and wear resistance^42,43^. Therefore, in this section, surface topography (surface roughness and surface waviness) is examined and discussed thoroughly.

**Figure 5:**
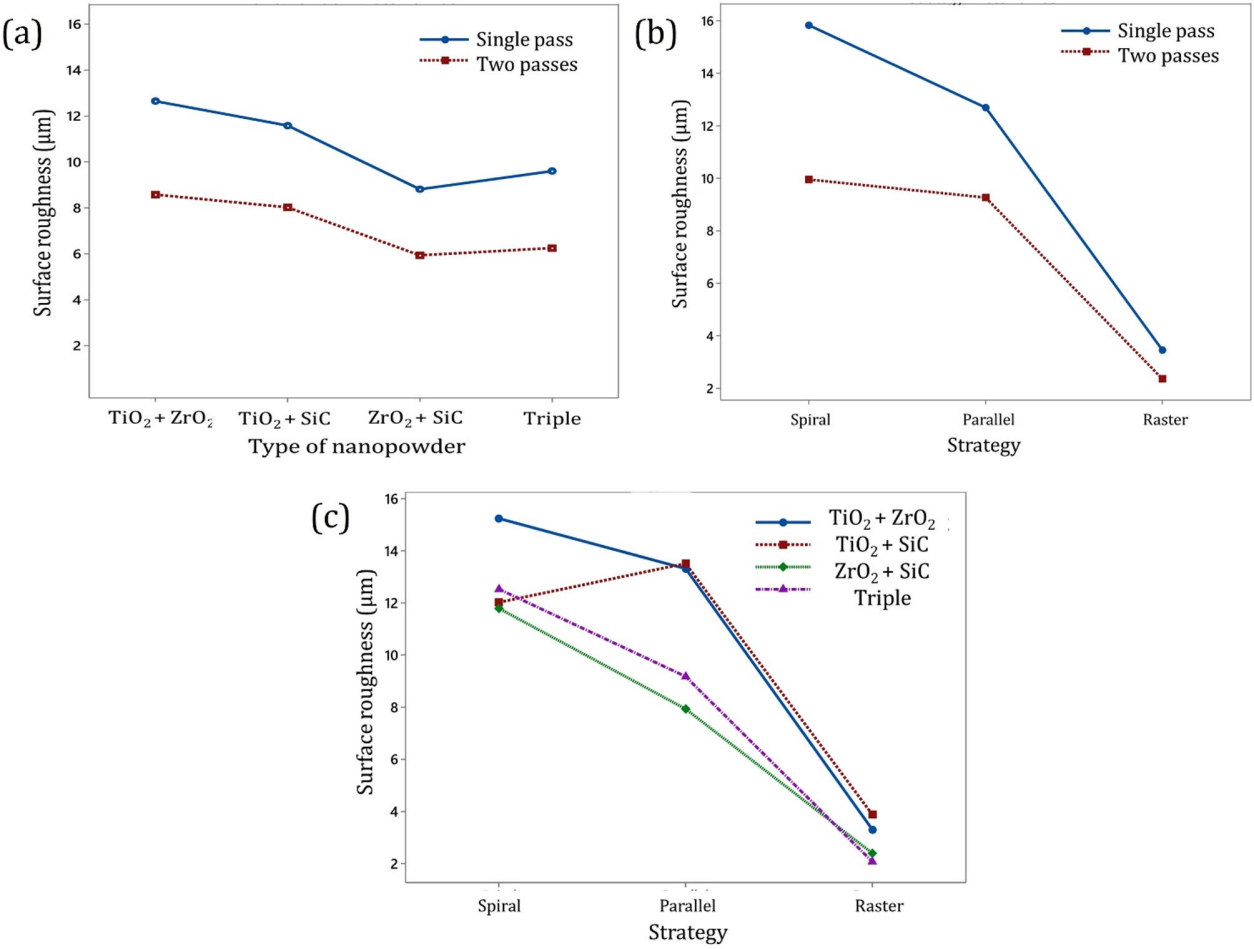
Interaction of a nanopowders and pass number, (**b**) strategy and pass number, and c strategy and nanopowders on surface roughness (R_a_) of hybrid nanocomposites.

Figure 5a and b show the pass number effect on AA6061 nanocomposite with different tool paths and various reinforcements. The surface processing with a single pass presented a higher arithmetic average surface value (R_a_) and undesirable surface quality. In other words, surface roughness decreased with increasing the pass numbers. The agglomeration and insufficient mixing of nanoparticles in the base metal and matrix have produced rougher surface and waviness in all types of the surface of AA6061 hybrid nanocomposites with one pass^44^. In addition, with the multi-pass number, the surface roughness and waviness decreased due to asperities formation and the nanoparticle fragmentation in the base metal. Also, the homogeneous distribution of the nanoparticles in the base metal during FSP is the main reason for asperities formation, and increasing the pass number improves surface quality due to the homogeneous distribution of the nanoparticles.

With increased heat generation during friction stir processing, the surface material becomes soft and obtains the proper surface roughness. Besides, the nanopowders in the aluminum base metal improve the surface roughness compared to the nonreinforced matrix^45^. In addition, Fig. 5a and c show each nanoparticle has specific characteristics that are effective on surface roughness. The surface roughness values decreased due to the addition of the nanoparticles, which smooth valleys and peaks. Also, the addition of nanoparticles causes a more suitable metallurgical bonding with the base metal because of the plasticized material flow stress during the FSP^22^. Results revealed that surface roughness was higher in compounds of nanoparticles with TiO_2_. On the contrary, the compounds of nanoparticles with ZrO_2_ had better surface roughness. Besides, the surface roughness of the hybrid nanocomposite with TiO_2_ + ZrO_2_ is higher than the surface roughness of the hybrid nanocomposite with SiC + ZrO_2_, which is due to forming a tribo layer for solid lubrication of multiple grooves formed on the surface during FSP^46,47^.

**Figure 6:**
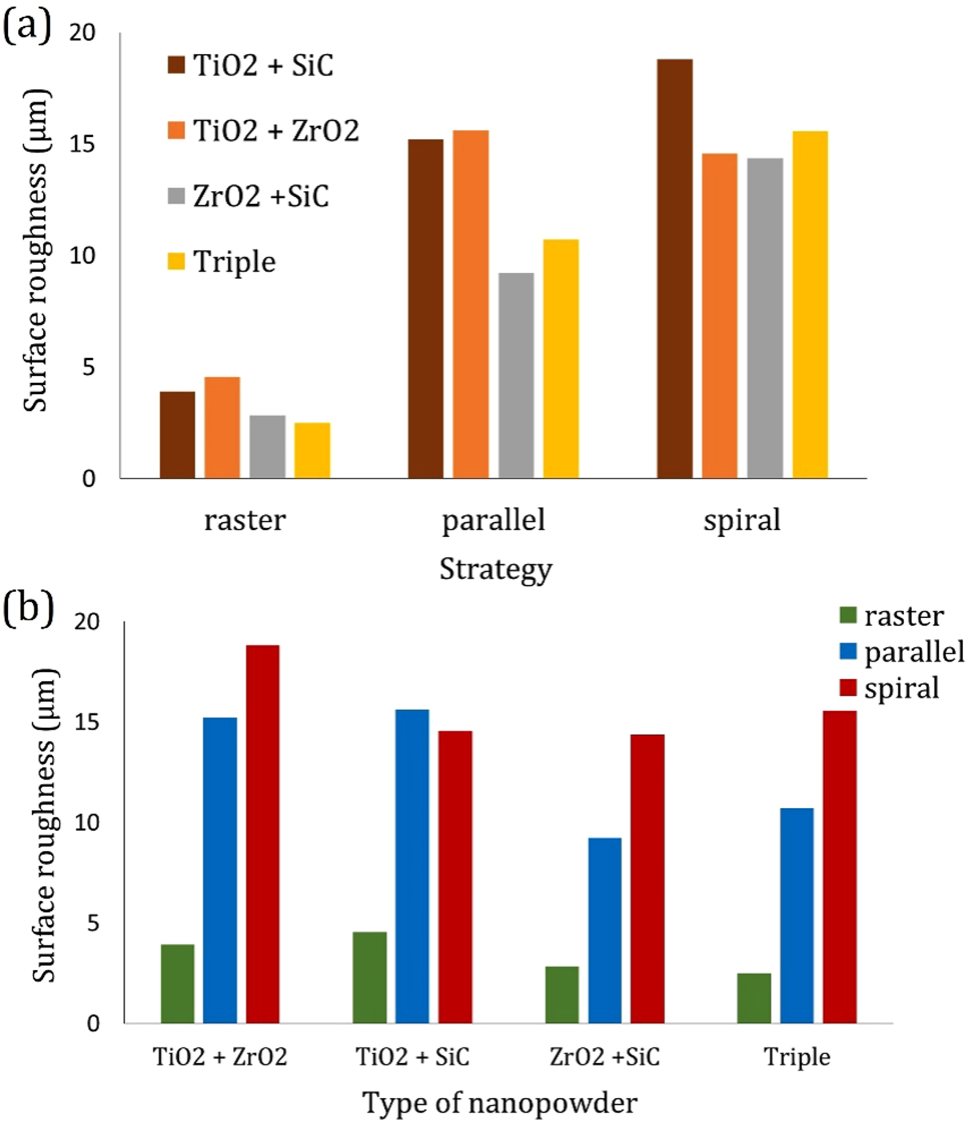
Overall comparison of the surface roughness in different a FSP strategies, and b reinforces.

The strategy generally used in FSP is the parallel path one. The parallel pass strategy is simple to generate, but this path is discontinuous. Furthermore, there is adequate time for the cooling in the revolution period, hence decreasing the tool wear. On the other hand, with the raster and spiral strategy, the tool path is continuous and appropriate for high-speed manufacturing. The continuous movement of the FSP tool with the zone area is beneficial for some reasons. In parallel strategy for providing extensive surface composite, periodic entering, and exiting strategy can create unfavorable stresses on the FSP tool or generate vibration and chatter. Therefore, the raster strategy could be employed rather than performing several parallel ones. The zone below the FSP tool and the region between the thermomechanical affected zone (TMAZ) and the stir zone (SZ) is an effective zone during friction stir processing. Thus, there are various properties from the advancing side (AS) to the retreating side (RS) due to the deformation zone. In the parallel pass, the advancing side is replaced with the retreating side in the processed material. Therefore, the surface roughness of the hybrid nanocomposite at the spiral strategy was higher than the parallel pass (Figs. 5b, c, and 6). Figure 6 shows an overall comparison of the surface roughness in different FSP strategies and reinforces. The result indicates the best surface roughness of aluminum surface nanocomposites is measured to be 1.63 µm during FSP with two passes for triple compound nanopowder at raster strategy. Moreover, the maximum surface roughness of aluminum surface nanocomposites is obtained to be 18.81 µm during FSP with a single pass for TiO_2_ + ZrO_2_ compound nanopowder at spiral strategy.

The hybrid nanocomposites are ultra-fine grain (UFG) structures due to severe plastic deformation (SPD)^48^. In the FSP, nanopowder is smashed into smaller fragments that will function as a barrier to the grain boundaries motion that reduces the grain size^49,50^. The surface topography and surface morphology of hybrid nanocomposite with ZrO_2_ + SiC reinforcement is better than the morphology of hybrid nanocomposite with TiO_2_ + SiC reinforcement which could be attributed to the fact that ZrO_2_ nanopowder tends to be smashed into smaller fragments than TiO_2_ nanopowder.

**Figure 7:**
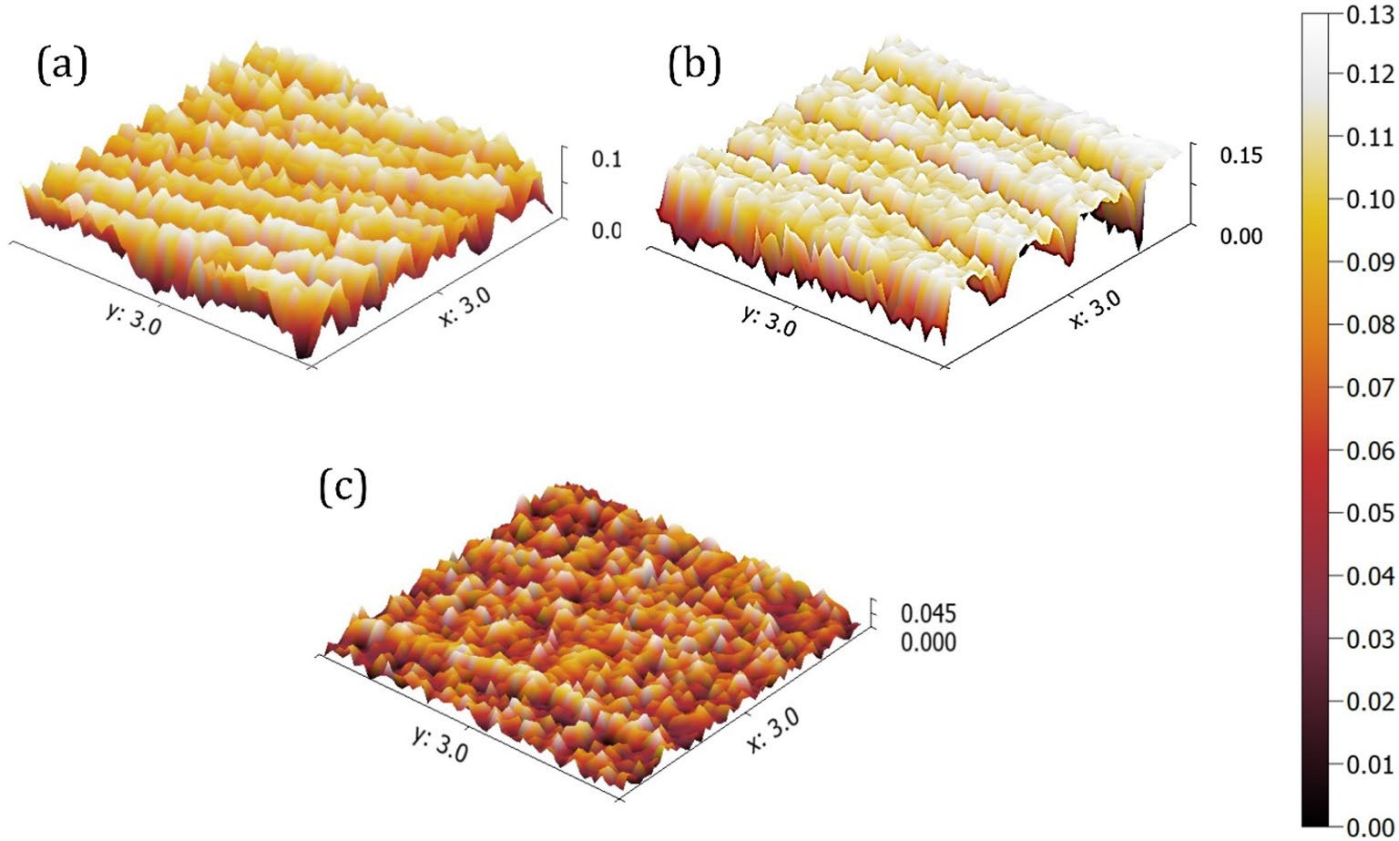
Surface topography by scanning probe methods in different process conditions: (**a**) spiral, (**b**) parallel, and (**c**) raster tool path.

Figure 7 shows the three-dimensional (3D) surface morphology of surface hybrid nanocomposite under different FSP strategies captured by AFM. Surface topography shows that the surface composites fabricated by the raster strategy were nearly symmetrical and smooth, while surface composites fabricated by the spiral strategy were irregular.

**Figure 8:**
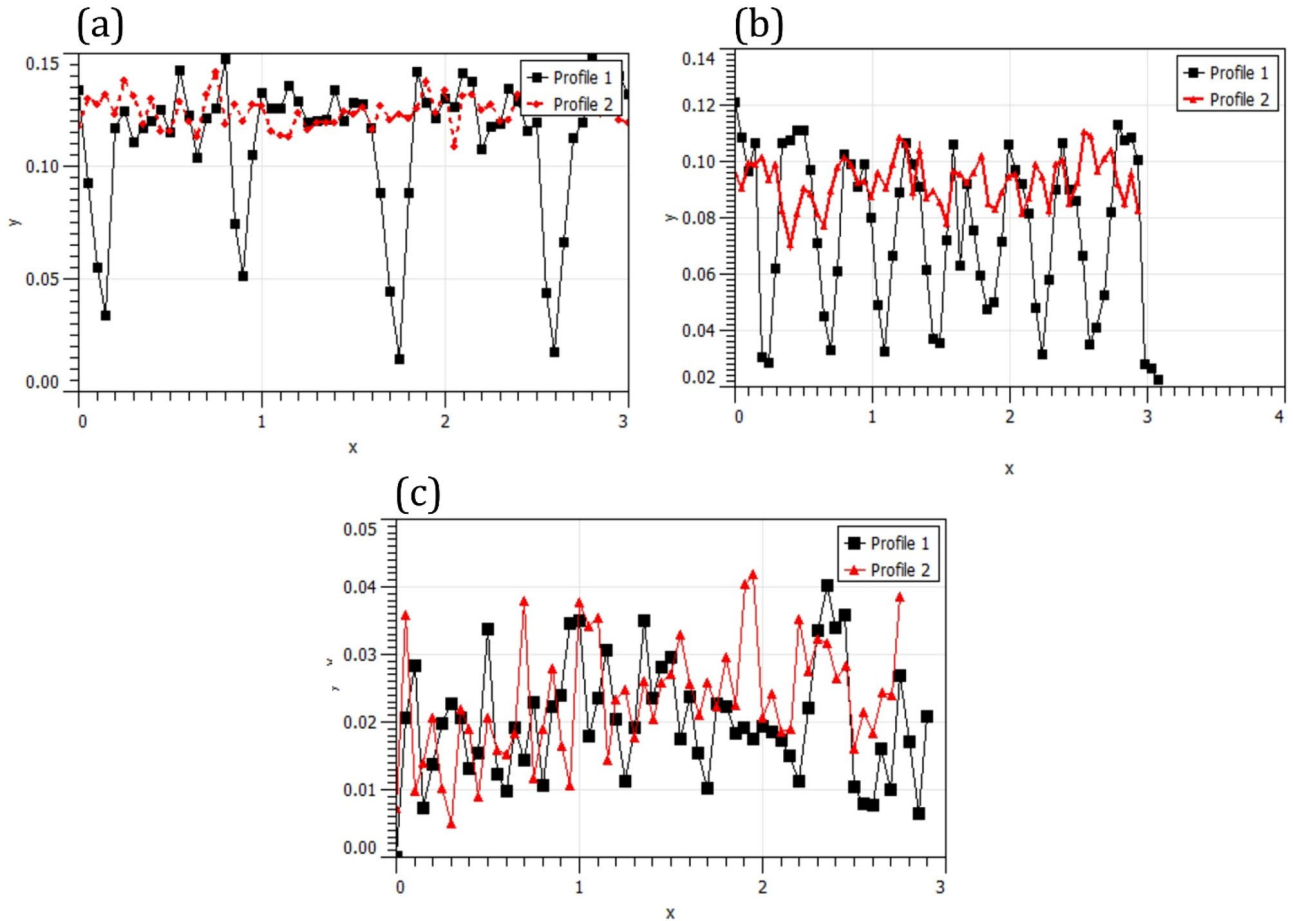
Surface profile in different process conditions: (**a**) spiral, (**b**) parallel, and (**c**) raster tool path.

Figure 8 shows the 2D surface profile of surface nanocomposite fabricated by different strategies. The raster strategy decreased the surface roughness and waviness. In Fig. 8, two profiles show the roughness of the surface nanocomposites in the longitudinal (profile 1) and transverse (profile 2) directions. Figure 8a, b show the longitudinal profile and transverse profile are different for spiral and parallel paths. In addition, the surface roughness profile in the transverse direction has a more favorable condition than the longitudinal profile. On the contrary, the raster FSP strategy profile is in a shorter range and has more suitable symmetry. Also, the roughness profile of the raster tool path had a similar trend in both the transverse and longitudinal directions.

**Figure 9:**
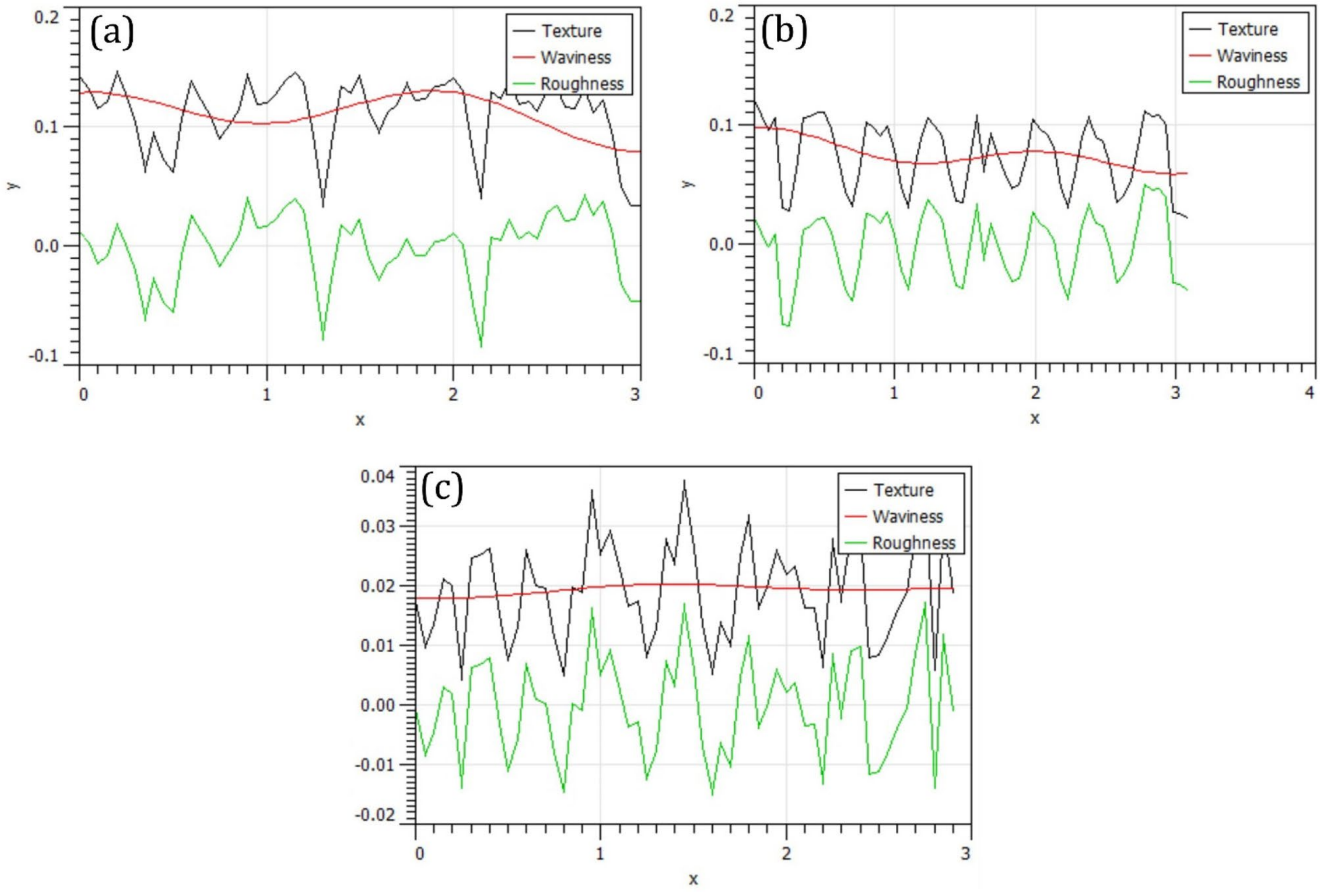
Surface texture and waviness in different process conditions: (**a**) spiral, (**b**) parallel, and **c** raster tool path.

Figure 9 shows surface texture of surface nanocomposite produced by various strategies. The waviness of the raster strategy is much less than the parallel and spiral strategies. Moreover, the range of waviness and texture is much smaller than parallel and spiral ones. As the height of valleys and peaks decreases, the distance between peak and valley also decreases and creates a finished surface with proper arithmetic average surface value (R_a_)^51–53^. Therefore, the surface roughness values decreased due to smooth valleys and peaks during the raster strategy.

### Metallurgical evaluation

The material flow on the advancing side is unlike on the retreating side. The maximum grain size is obtained with proper arithmetic average surface value (R_a_). On the contrary, the minimum grain size is obtained with the least arithmetic average surface value. Consequently, grain size in the friction zone is decreased when the surface roughness value is reduced. The grain size of the processing region and grain refining decreases when heat generation increases^54^. Accordingly, an increase in heat generation triggers the proper surface roughness value^55,56^.

The FSP is an appropriate and effective process that delivers ultra-fine grain (UFG) materials by employing severe plastic deformation in the nugget zone, leading to microstructure modification. Besides, in this process, the deformation and heat generation cause the aluminum matrix to flow at a semi-solid temperature (under the melting point). The stress and temperature are lower in the process area for the retreating side (RS) rather than the advancing side (AS). This difference is due to rotation and the movement of the FSP tool which is opposite on the RS and the same on the AS.

FSP strategy can also determine grain size and microstructure. Parallel, raster, and spiral processing have various effects on microstructural that are attributed to thermal cycle changes. In the parallel tool path, the fabricated surface composite is cooled after each path with discontinuous movement. Therefore, overlapping of passes in a comprehensive surface can be achieved. On the other hand, the raster and the spiral FSP strategies are continuous processes without providing adequate cooling time.

This approach is one of the hot deformation procedures in which dynamic recovery (DRV) and dynamic recrystallization (DRX) appeared during the process. Therefore, new grain growth occurred due to the continuous dynamic recrystallization (CDRX) and discontinuous dynamic recrystallization. These mechanisms make aluminum microstructure refinement.

**Figure 10:**
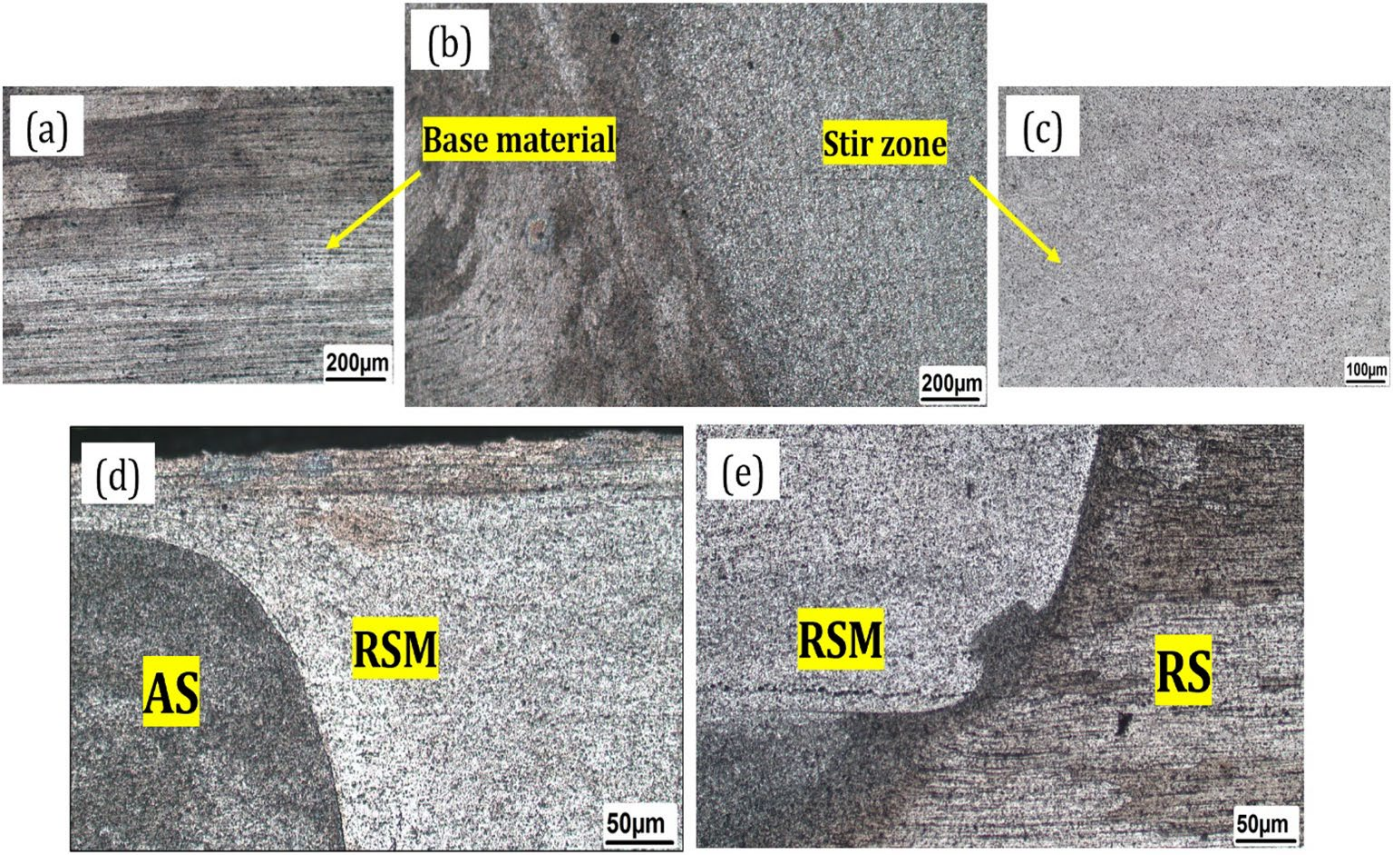
Macro images of cross section of the AA6061 hybrid nanocomposite: (**a**) base material area, (**b**) interphase of FSP, (**c**) stir zone region, d advancing side (AS), and **e** retreating side (RS).

**Figure 11:**
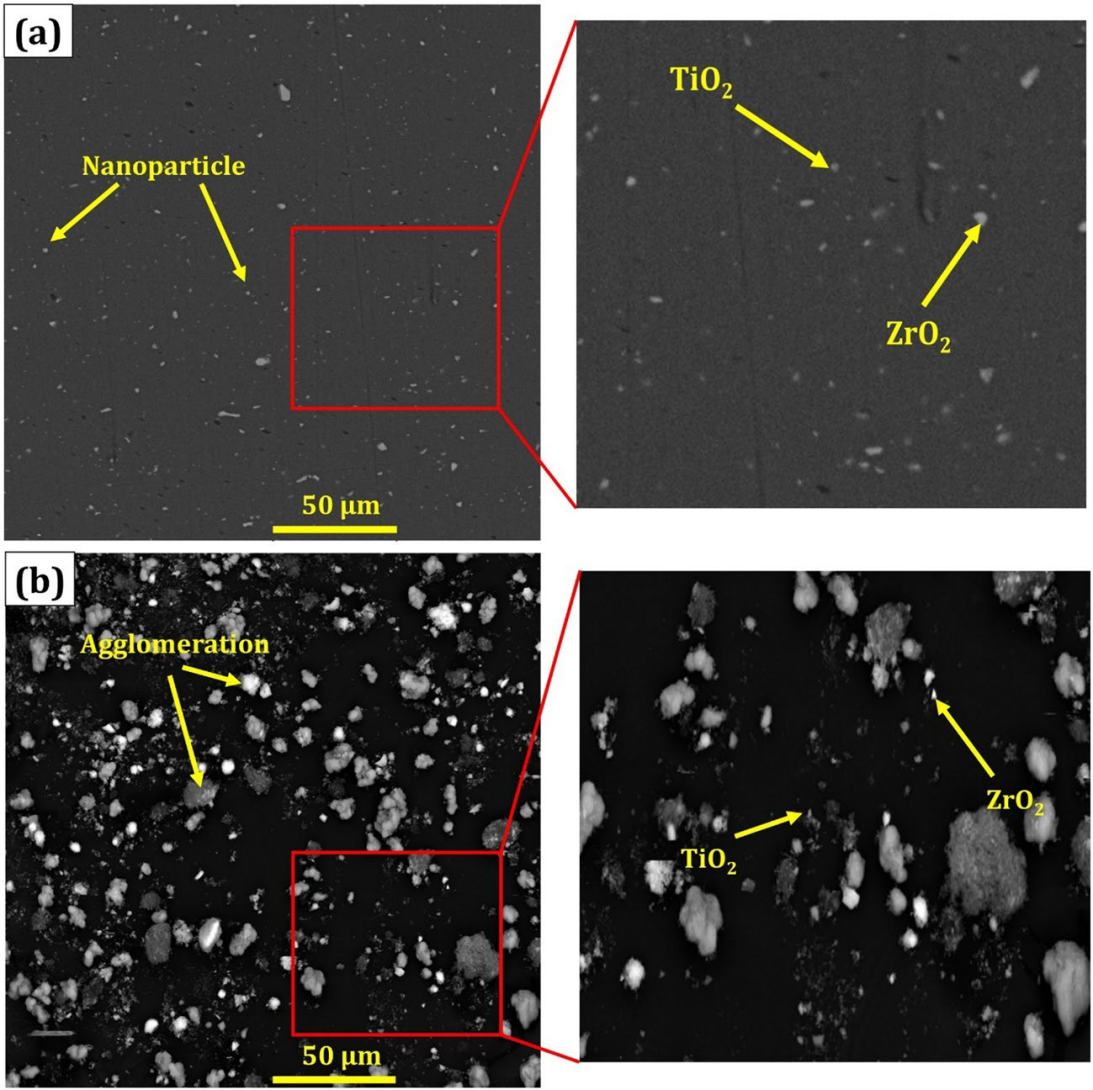
SEM images of the AA6061 TiO_2_–ZrO_2_ hybrid nanocomposite microstructure in different process conditions: a parallel and b spiral strategy.

**Figure 12:**
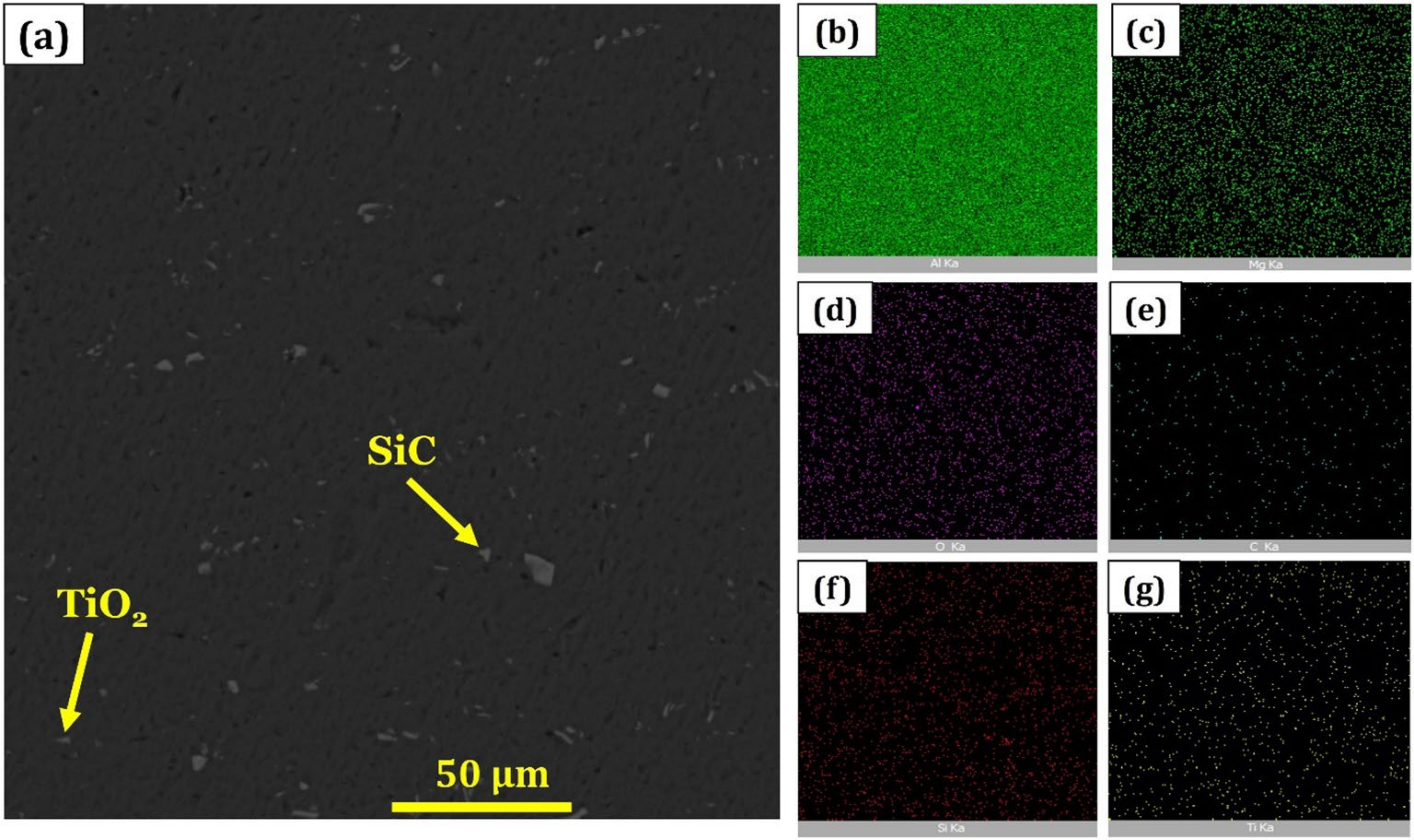
SEM images of microstructure of raster strategy from the AA6061 TiO_2_-SiC hybrid nanocomposite as well as the related EDS analysis maps for different elements of (**b**) Al, (**c**) Mg, (**d**) O, (**e**) C, (**f**) Si, and (**g**) Ti.

There is a rotational shear material (RSM) at the stir zone. The widespread RSM region can be seen on the advancing side, but instead, this region is found only on the retreating side in the lower part of the stir zone (Fig. 10). Besides, in RSM, nanopowders and nanoparticles are distributed in the metal matrix homogeneously, and outside of the RSM area, the microstructure is less deformed, especially on the retreating side^57^. Therefore, in parallel FSP strategy with displacement of the advancing side (AS) and the retreating side (RS) in the processing, nanoparticles are distributed homogeneously and very finely (Figs. 11 and 12).

Moreover, the homogeneous and fine distribution of the nanoparticles prevent grain growth by pinning the boundaries of the grain. Thus, in the parallel tool path of FSP, significant ultra-fine size is attained due to arrested grain growth and more pinning action of the nanoparticles. In addition, based on the results, dislocations, and sub-boundaries entered the process area and were mixed instead of restricted to their slip planes due to stacking-fault energy (SFE). Moreover, the microstructure with small sub grains demonstrates severe dynamic recovery during FSP.

The successive friction stir processing strategy can ensure selective material properties. Thus, microstructure investigation during the different tool paths and FSP strategy is crucial. The zone below the FSP tool and the region between the thermomechanical affected zone and the stir zone is an effective zone during FSP. Therefore, there are microstructure gradients from the advancing side to the retreating side due to the deformation zone. Figure 11 shows a microstructure of nanocomposite with magnified images. In the parallel pass, a homogeneous distribution of nanoparticles was observed. This homogeneous distribution was due to the displacement of the advancing side with the retreating side in the processing. In contrast, the agglomeration and insufficient mixing of nanoparticles in the base metal and matrix was observed in the spiral strategy which could be attributed to the displacement of the advancing side with a retreating side, and this replacement decreases the stir zone. This situation may create strain localization, reduced ductility, and an incomplete overlap in the stir zone.

Scanning electron microscope (SEM) and mapping of elements were utilized to investigate the composition, distribution, and morphology of hybrid nanopowders in base metal which play a vital role in physical and mechanical properties of manufactured nanocomposite. Although the addition of nanoparticles results in grain refinement and improved mechanical properties, the possibility of facing challenges such as agglomeration during the addition of hybrid nanoparticles to the metal matrix remains. These challenges could be related to various plasticization degrees and various flow features of different nanopowders during FSP. The plasticization degree of TiO_2_–ZrO_2_ hybrid nanocomposite with spiral strategy was not adequate for the nanopowder dispersion compared to TiO_2_–ZrO_2_ hybrid nanocomposite with raster and parallel strategy.

All types of nanoparticles are observable in the hybrid nanocomposite shown in Figs. 11 and 12, indicating that the hybrid nanopowder addition can improve the aluminum base metal mechanical properties when the distribution of nanoparticles is desirable on the surface of the matrix.

Also, SEM images of microstructure from the hybrid nanocomposite created by the raster strategy with the related EDS analysis mapping are shown in Fig. 12. Based on the titanium and silicon map in Fig. 12, an excellent distribution of precipitation can be seen by the base metal and matrix grain structure. Also, in the parallel pass strategy, the distribution of nanoparticles is better than the raster strategy. This is because of heat generation and more reasonable reinforcement mixing. The raster strategy may cause strain localization and the microstructure gradient to change so that the structure of each area is different from the other one^15,17^.

### Mechanical properties

The microhardness of all hybrid nanocomposite samples was enhanced after the FSP in the stir zone, whereas the aluminum matrix displayed a mean microhardness of 61.18 HV. This continuous improvement of stir zone microhardness could be attributed to a combination of secondary phase enhancing nanoparticles and grain refinement base on a micro-mechanical mechanism.

**Figure 13:**
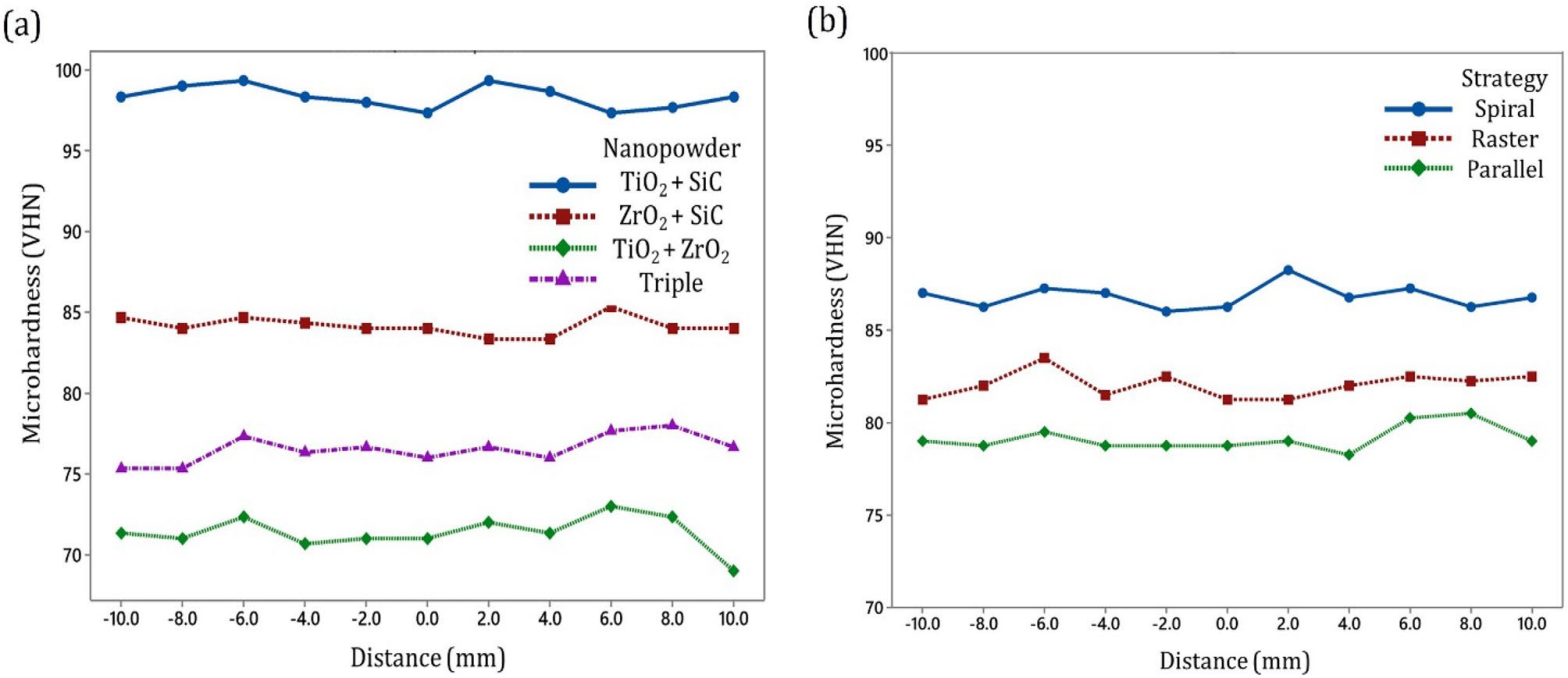
Hybrid nanocomposites mean microhardness profiles at NZ with different a nanopowders, and b strategies.

The nanopowder type influenced microhardness by resisting to plastic deformation which is due to the obstacles of reinforcement to the dislocation motion^58^. The overall of Fig. 13 shows that nanocomposites with hybrid nanopowders greatly enriche the microhardness behavior during FSP for high-performance applications.

Also, Fig. 13a illustrates the hybrid nanocomposite micro-hardness profiles at NZ with different nanopowders, including TiO_2_ + SiC, ZrO_2_ + SiC, TiO_2_ + ZrO_2_, and triple. As can be seen, the microhardness in the stir zones of the samples increased due to the presence of nanopowders. The mean microhardness of the TiO_2_ + SiC, ZrO_2_ + SiC, TiO_2_ + ZrO_2_, and triple obtained to be ~ 98.3, 84.1, 71.6, and 76.5 VH, respectively. In addition, in all four different hybrid nanocomposites, the microhardness value is increased due to secondary phase incorporation mechanisms^59,60^. Also, the variation of microhardness profiles can be attributed to the onion ring flow of nanoparticles intermixing and distribution^61^.

The mechanical properties of silicon carbide nanoparticles are greater than titanium oxide nanoparticles since a higher microhardness value is attained owing to the hard nature of the silicon carbide phase. Besides, the microhardness value of the hybrid nanocomposite with titanium oxide compound was higher than the microhardness value of the hybrid nanocomposite with zirconium oxide compound due to the strengthening effect of titanium oxide. Therefore, the composition of TiO_2_ and SiC leads to a more significant microhardness value in hybrid nanocomposite. Furthermore, the composition of TiO_2_ and SiC appears to be more restrictive to dislocation movement and can improve the grain boundary strength compared to the other compositions. Accordingly, grain boundaries are more reinforced, and a dispersion-strengthening process is more dominant compared to the other compositions, indicating that the nanopowder selection is critical in determining the mechanical properties of the hybrid nanocomposite produced by FSP.

**Figure 14:**
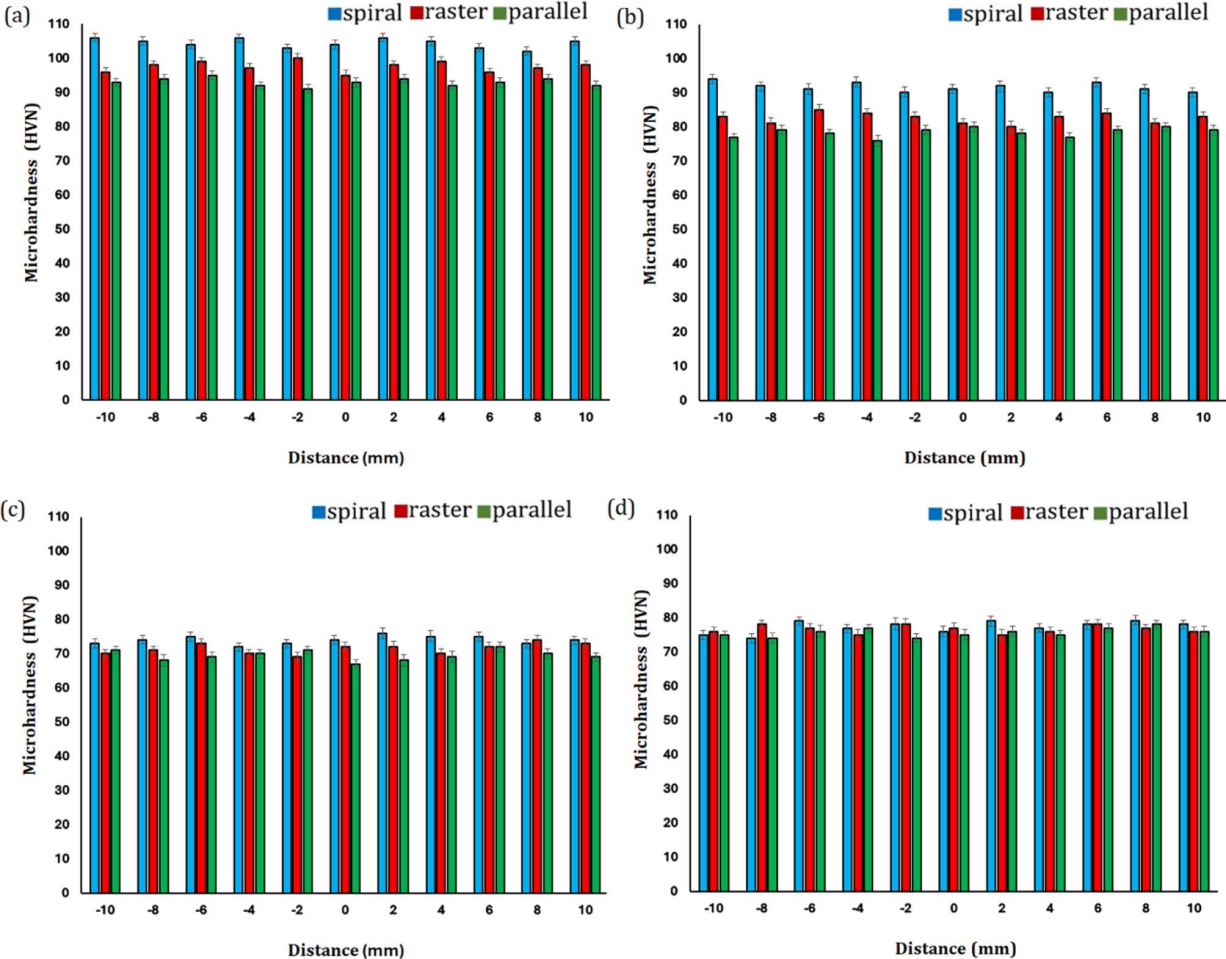
Overall comparison of the microhardness in different FSP strategies and reinforces: (**a**) TiO_2_–SiC hybrid nanocomposite, (**b**) ZrO_2_–SiC hybrid nanocomposite, (**c**) TiO_2_–ZrO_2_ hybrid nanocomposite, and (**d**) triple nanocomposite.

The mean microhardness profiles of hybrid nanocomposites with different strategies and tool paths are shown in Fig. 13b. As can be seen, the mean microhardness in the spiral, raster, and parallel pass strategies were obtained to be ~ 87, 82, and 79 VH, respectively. The mean microhardness profiles of hybrid nanocomposites produced during FSP created proper value compared to base material. As shown, the mean microhardness of nanocomposites at the spiral strategy exhibited higher than the parallel pass and raster strategy. The primary reason for this progress was due to the higher density of dislocation. Furthermore, in the surface nanocomposite produced by the spiral strategy, clusters of reinforcement are smashed better into smaller particles. Figure 14 shows an Overall comparison of the microhardness in different FSP strategies and reinforces. The result indicates the best microhardness of aluminum is measured to be 106 VH during FSP with TiO_2_ + SiC nanopowder at spiral strategy. Also, the minimum microhardness of aluminum surface nanocomposites is obtained to be 67 VH during FSP with TiO_2_ + ZrO_2_ compound nanopowder at parallel strategy.

## Conclusion

In the current article, raster, spiral, and parallel passes of FSP strategies were evaluated for the fabrication of HMMC. Also, this article concentrated on exploring the effects of the surface integrity of Al6061 hybrid nanocomposite, including surface roughness, surface topography, microstructure, EDS analysis, and microhardness. The main investigation results are summarized as follows:

- The surface processing with a single pass presented a higher surface roughness (R_a_) and surface waviness compared to two FSP pass numbers. In other words, surface roughness decreased with the pass number increasing.
- The raster strategy improved the surface roughness and waviness. The roughness profile of the raster tool path had a similar trend in both the transverse and longitudinal directions. Also, the surface roughness of the hybrid nanocomposite at the spiral strategy was higher than the parallel.
- The surface roughness was higher in compounds of nanoparticles with TiO_2_. On the contrary, the compounds of nanoparticles with ZrO_2_ had better surface roughness.
- SEM images demonstrated a fine microstructure obtained by the parallel FSP and raster strategy due to the homogeneous distribution of nanoparticles in the base metal.
- The microhardness value of the hybrid nanocomposite with TiO_2_ and Sic compound was higher than the microhardness value of the hybrid nanocomposite with ZrO_2_ compound which could attributed to the strengthening effect of titanium oxide and silicon carbide. The microhardness increased due to the presence of nanopowders. The mean microhardness of the TiO_2_ + SiC, ZrO_2_ + SiC, TiO_2_ + ZrO_2_, and triple obtained to be ~ 98.3, 84.1, 71.6, and 76.5, respectively.

## Data Availability

All data generated or analyzed during this study are included in this published article.
