# Author Correction: Evaluating the influence of various friction stir processing strategies on surface integrity of hybrid nanocomposite Al6061

**DOI:** 10.1038/s41598-024-83261-2

**Published:** 2024-12-23

**Authors:** Navid Molla Ramezani, Behnam Davoodi

**Affiliations:** School of Mechanical Engineering, Iran University of Science and Technology, Tehran, Iran

Correction to: *Scientific Reports* 10.1038/s41598-024-58714-3, published online 05 April 2024

The original version of this Article contained errors.

A citation to Reference 3 was omitted from the legend of Figure 2.

“The friction stir processing: (**a**) experimental setup, (**b**) schematic of the process, and (**c**) FSP tool.”

now reads:

“The friction stir processing: (**a**) experimental setup, (**b**) schematic of the process, adapted under license from^3^ and (**c**) FSP tool.”

Furthermore, a reference was omitted from the legend of Figure 4. The reference is listed below as Reference 39:

39. Sekban, D.M., Aktarer, S.M. & Purcek, G. Friction Stir Welding of Low-Carbon Shipbuilding Steel Plates: Microstructure, Mechanical Properties, and Corrosion Behavior. *Metall Mater Trans A*. **50**, 4127–4140 (2019).

As a result, the legend of Figure 4,

“Schematics illustration of sample locations for determining surface roughness, surface topography, microstructure, and microhardness.”

now reads:

“Schematics illustration of sample locations for determining surface roughness, surface topography, microstructure, and microhardness. Adapted under license from^39^.”

All subsequent references have been renumbered.

The original Article has been corrected.

